# Developing Immunity Testing for SARS-CoV-2 and Other Novel Vaccines—Correlates of Immunogenicity Parameters with Protection

**DOI:** 10.3390/v14020196

**Published:** 2022-01-20

**Authors:** Zoltan Vajo

**Affiliations:** Department of Family Practice, Semmelweis University, 1085 Budapest, Hungary; zoltanvajo@gmail.com

During the assessment and licensing of novel vaccines, as well as post licensure follow up, it is critical to have reliable immunogenicity testing methods that relate well to real life protection. The concept is straightforward, but it can be more complicated than it seems. With influenza, for instance, we believed for decades that a 1:40 titer of hemagglutinin inhibition assays corresponds to approximately 50% protection against the development of disease [1]. Therefore, most licensing criteria were based on that simple parameter [2]. However, the need for more reliable immunogenicity criteria, as well as the complex nature of the issue, became apparent again during preparation for the potential pandemic threat by influenza A H5N1 in 2006, when great intra- and inter-laboratory variations were seen for hemagglutinin inhibition, as well as microneutralization tests [3]. Thus, the need for a centralized standard was acknowledged by the WHO and NIBSC. 

The complexity of immunogenicity markers could also explain the resistance of older subjects to severe disease when the influenza A H1N1 virus subtype reappeared in 1977 and again in 2009/10, when mainly people younger than 40 years old were susceptible, although a number of resistant subjects had no detectable antibodies. Since then, the European Medicines Agency has recognized the issue and changed its licensing criteria for novel seasonal influenza vaccines, as well as its yearly licensing requirements [4]. The new guideline adopted a more diversified approach to the measurement and reporting of the immune response to influenza vaccines and sets a requirement to conduct clinical outcome trials in young children.

In the case of SARS-CoV-2, this remains a critical issue, as many different antibody tests have been developed urgently, however, with yet unknown correlation to real world protection. There is already evidence for at least some association, even between the level of artificial units of antibodies and vaccine efficacy, but we are far from final conclusions [5]. Currently, even less is known about the role of cellular immunity in vaccine efficacy against COVID-19. Therefore, collaboration among different laboratories and health authorities is essential to establish standards and to reduce inter laboratory variations, as well as to establish immunogenicity correlates that can be used for licensing criteria as well as patient care. New vaccines are being developed more and more rapidly, and hence, this subject is as important as ever. 

In this Special Issue, we publish papers discussing the development of reliable immunogenicity assays and how they correlate with the real-world protection of SARS-CoV-2 and other novel vaccines. Ongoing cooperation of academic institutes, manufacturers, public health officials and regulators to address these challenges are needed to develop optimal tools to evaluate and monitor the performance of current and future vaccines.
